# Supplementary material for: Providing brief information on clinical trials in appropriate formats may improve impressions and willingness to participate among socioeconomically disadvantaged people in France
Source: PLoS One. 2025 Jul 29;20(7):e0329288. doi: 10.1371/journal.pone.0329288 (PMC12306746; doi:10.1371/journal.pone.0329288)
Supplement: S1 Fig — shows the evolution of the median score of willingness to participate in clinical trials after reading the information note on clinical trials (median from 5 (3, 7) to 5 (4, 7); p < 0.001, effect size r = 0.20). (PDF) [file pone.0329288.s001.pdf]

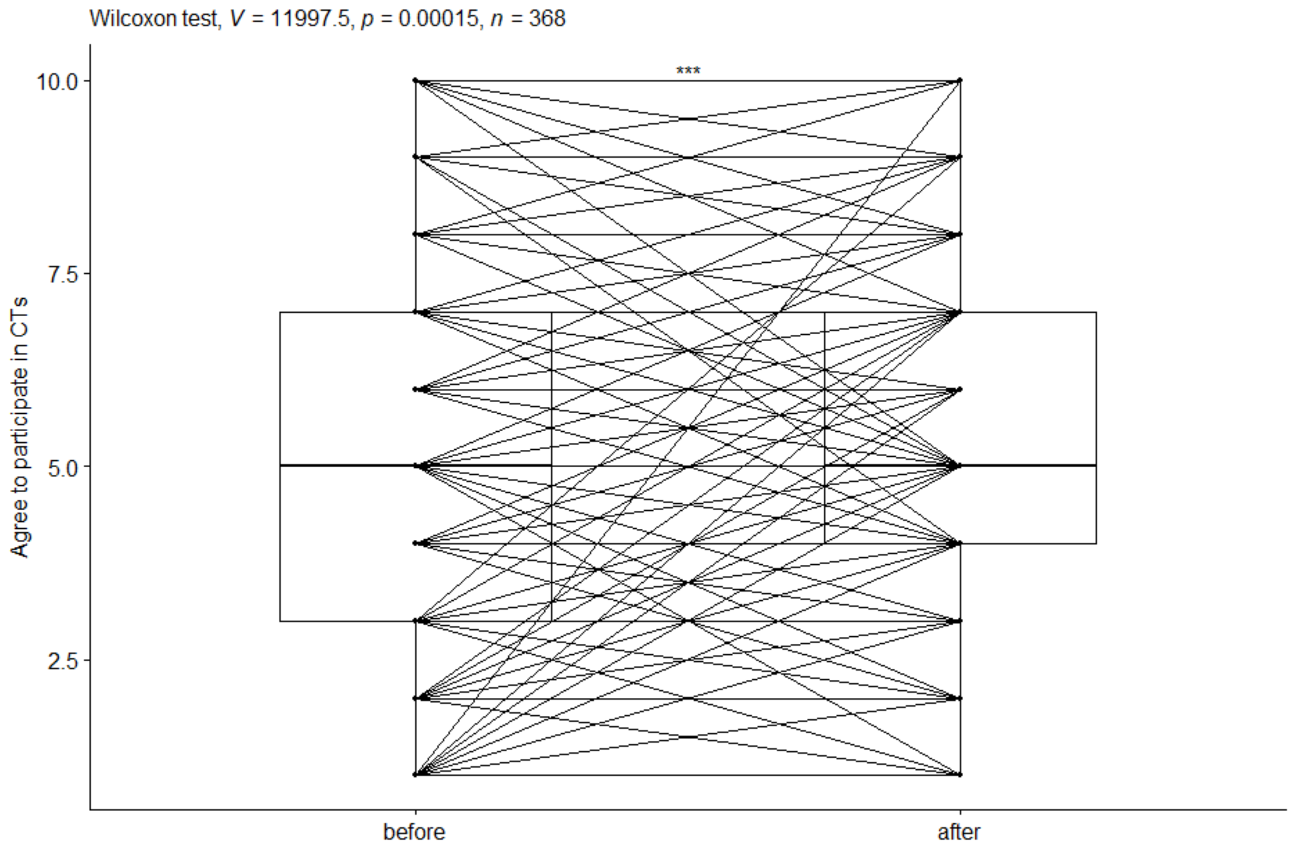

S1 Fig. Evolution of the 'hypothetical willingness to participate in a clinical trial' score after reading the brief information note about clinical trials for the whole sample. S1 Fig shows the evolution of the median score of willingness to participate in clinical trials after reading the information note on clinical trials (median from 5 (3, 7) to 5 (4, 7); $p < 0.001$, effect size $r = 0.20$)
