# Supplementary material for: Providing brief information on clinical trials in appropriate formats may improve impressions and willingness to participate among socioeconomically disadvantaged people in France
Source: PLoS One. 2025 Jul 29;20(7):e0329288. doi: 10.1371/journal.pone.0329288 (PMC12306746; doi:10.1371/journal.pone.0329288)
Supplement: S1 Table — The evolution of participants’ general impression of clinical trials (CTs) according to the type of information received was measured according to participants’ health literacy and numeracy level. S1 Table shows the evolution of the general impression of CTs in participants with adequate literacy versus those with limited literacy, and those with high subjective numeracy versus those with low subjective numeracy. (PDF) [file pone.0329288.s002.pdf]

S1 Table. Evolution of participants' general impression on CTs score according to their health literacy, numeracy, and the format of information on CTs provided.

	**Textual information**				**Tabular information**			
	Median general impression of CTs (0-10)				Median general impression of CTs (0-10)			
	Before	After	Effect size	p-value	Before	After	Effect size	p-value
Adequate HL	5	7	0.56	< **0.001**	5.5	7	0.51	< **0.001**
Limited HL	5	6	0.41	**0.002**	5	6	0.41	< **0.001**
Higher Numeracy	6	7	0.37	**0.006**	6	7	0.53	< **0.001**
Lower Numeracy	5	6	0.59	< **0.001**	5	6	0.39	< **0.001**

CT: clinical trial, HL: functional health literacy

The evolution of participants' general impression of clinical trials (CTs) according to the type of information received was measured according to participants' health literacy and numeracy level. **S1 Table** shows the evolution of the general impression of CTs in participants with adequate literacy versus those with limited literacy, and those with high subjective numeracy versus those with low subjective numeracy.
