# Supplementary material for: Providing brief information on clinical trials in appropriate formats may improve impressions and willingness to participate among socioeconomically disadvantaged people in France
Source: PLoS One. 2025 Jul 29;20(7):e0329288. doi: 10.1371/journal.pone.0329288 (PMC12306746; doi:10.1371/journal.pone.0329288)
Supplement: S2 Table — presents two linear regression models. The variable of interest in the first is the general impression of clinical trials before reading the brief information note, while the second model presents the variables associated with general impression of clinical trials after reading the information note. The third model presents the variables associated with the change in impression after reading the information note. (PDF) [file pone.0329288.s003.pdf]

S2 Table. Linear regression models on participants' general impression of clinical trials (before and after reading brief information note).

Characteristic	General impression before		General impression after		Change in impression	
	β [CI95%]	p-value	β [CI95%]	p-value	β [CI95%]	p-value
Trust in French medicine	0.86 [0.64, 1.1]	**<0.001**	1.1 [0.94, 1.3]	**<0.001**	0.27 [0.09, 0.45]	**0.003**
Age (years)	0.00 [-0.01, 0.02]	0.8	-0.01 [-0.02, 0.00]	0.14	-0.01 [-0.02, 0.00]	0.10
Sex (male)	-0.09 [-0.50, 0.32]	0.7	-0.02 [-0.38, 0.34]	>0.9	0.09 [-0.24, 0.42]	0.6
Household income	-0.09 [-0.33, 0.16]	0.5	0.07 [-0.14, 0.29]	0.5	0.18 [-0.02, 0.38]	0.071
Education level (Higher than high-school graduate)	0.06 [-0.35, 0.48]	0.8	0.06 [-0.31, 0.42]	0.8	0.04 [-0.30, 0.38]	0.8
French was not mother tongue	-0.55 [-1.7, 0.60]	0.3	-0.83 [-1.8, 0.15]	0.10	-0.15 [-1.1, 0.78]	0.7
Inadequate HL	0.28 [-0.12, 0.67]	0.2	-0.03 [-0.38, 0.32]	0.9	-0.29 [-0.61, 0.04]	0.082
Group of randomization (Group CT-1)	NA	NA	0.01 [-0.36, 0.37]	>0.9	-0.09 [-0.42, 0.25]	0.6

HL: functional health literacy

S2 Table presents three linear regression models. The variable of interest in the first is the general impression of clinical trials before reading the brief information note, while the second model presents the variables associated with general impression of clinical trials after reading the information note. The third model presents the variables associated with the change in impression after reading the information note.
