# Supplementary material for: Providing brief information on clinical trials in appropriate formats may improve impressions and willingness to participate among socioeconomically disadvantaged people in France
Source: PLoS One. 2025 Jul 29;20(7):e0329288. doi: 10.1371/journal.pone.0329288 (PMC12306746; doi:10.1371/journal.pone.0329288)
Supplement: S1 File — (PDF) [file pone.0329288.s004.pdf]

**S1 File. Information about clinical trials provided to participants.**

A brief information note on clinical trials was provided to survey participants in three formats (1, 2a, 2b). Format 1 was textual; formats 2a and 2b were both in tabular form. Participants were randomized into three groups, one for each format. The difference between groups 2a and 2b lay in the fact that format 2b had information about risks and constraints.

## Information about clinical trials – Format 1

Clinical trials are studies that aim to test new ways of treating, diagnosing, and preventing disease in volunteer participants. They help determine whether a new diagnostic test or treatment is safe and effective. Examples of treatments studied include new drugs, new surgical procedures, new techniques, and new ways of using or improving existing treatments. These trials cover thousands of different diseases.

After disease diagnosis, the benefits of participating in a clinical trial are:

- Receiving treatment several years before it becomes widely available.
- Receiving (in the majority of cases) the new treatment under study in addition to current standard treatment.
- Better management of disease symptoms and side effects and better general well-being.
- Better quality care and follow-up.

In most trials, one group (usually half of the participants) receives the new treatment in addition to the standard treatment, while a control group receives the current standard treatment. People who agree to participate in a clinical trial are free to leave it at any time. The costs of clinical trials are covered by national health insurance.

Clinical trials are essential for advancing medical knowledge. Almost all of the treatments used today were first tested and made available to patients through clinical trials.

## Information about clinical trials – Format 2a

| Frequently asked questions ↓ | Participation in a clinical trial | Standard care |
|---|---|---|
| **What will my treatment be?** | Patients (usually half) receive the new treatment, in the majority of cases in addition to the current standard treatment.<br>The rest are given the standard treatment, which is the best current treatment available. | All patients receive standard treatment. |
| **What are the benefits?** | • Receiving a new treatment several years before it becomes widely available. This treatment can better manage symptoms and side effects of the disease and improve general well-being.<br>• Improved quality care and follow-up.<br>• Advancing medical knowledge. Almost all the treatments used today were first tested and made available to patients through clinical trials. | The same benefits associated with standard clinical practice |
| **Are there any additional costs?** | The costs are covered by health insurance | No |
| **Can I change my mind?** | Participants can leave the trial at any time. | A reflection period allows you to decide whether or not to participate. |

## Information about clinical trials – Format 2b

| Frequently asked questions ↓ | Participation in a clinical trial | Standard care |
|---|---|---|
| **What will my treatment be?** | Patients (usually half) receive the new treatment, in the majority of cases in addition to the current standard treatment.<br>The rest are given the standard treatment, which is the best current treatment available. | All patients receive standard treatment. |
| **What are the benefits?** | • Receiving a new treatment several years before it becomes widely available. This treatment can better manage symptoms and side effects of the disease and improve general well-being.<br>• Improved quality care and follow-up.<br>• Advancing medical knowledge. Almost all the treatments used today were first tested and made available to patients through clinical trials. | The same benefits associated with standard clinical practice |
| **What are the risks?** | While the new treatment helps some patients, it may not work for everyone. When a treatment has only been previously tested on a limited number of patients, some rare side effects may not be identified. | The same benefits associated with standard clinical practice |
| **Are there any additional constraints?** | Participants must sign a consent form which provides all the information necessary to decide whether or not to participate. Participants frequently have more tests (e.g., blood tests or scans) and follow-up visits. | No |

**Note**

We initially developed an information table with six questions and answers, but it was not possible to include all six questions at once, as the text would have been significantly longer compared to the text provided to group 1. This is why we formed two subgroups, each with two common and two different questions, including specific information about the potential risks and burden of CT participation in group 2b.
