# Supplementary material for: Providing brief information on clinical trials in appropriate formats may improve impressions and willingness to participate among socioeconomically disadvantaged people in France
Source: PLoS One. 2025 Jul 29;20(7):e0329288. doi: 10.1371/journal.pone.0329288 (PMC12306746; doi:10.1371/journal.pone.0329288)
Supplement: Attachment — Submitted filename: PLOS_CT Information Sheet & Consent_Reviewer Comments.docx [file pone.0329288.s005.docx]

Reviewer Comments on the article titled “Providing brief information on clinical trials in the most appropriate format to socioeconomically disadvantaged people may improve their impression of and participation in these trials”

Lines 90 & 91, and lines 108-109 – The first objective of the study reads “to assess whether providing brief information on CTs improved the general impression of and willingness to participate in CTs in a sample of socioeconomically disadvantaged” whereas the exclusion criteria say “persons who reported that they had never heard of CTs in the survey questionnaire were 108 secondarily excluded from the analysis.” If people were not provided information, how are they expected to participate irrespective of the method of information? Please explain why this was given as an exclusion factor

Line 100, page 4 – Please provide brief information on what is meant by the IPSOS i-Say sampling panel

Lines 144-149, page 5 – The authors mention 3 groups, but there is no explanation on why there are 3 groups in the study, and what the 3 groups are. Please provide the rationale

Lines 145-147, page 5 – There is no explanation on why a tabular form was chosen for the information regarding CTs. What was the rationale behind choosing the tabular formats?

Lines 145-147, page 5 – Why are there two tabular formats, and why was it pooled later during the analysis?

Lines 160-162, page 6 – Why was group 1 excluded from the analysis? Please provide an explanation

Table 1, page 7 – While the study objective is to see whether there is an improved willingness to participate in CT and the general impression about the CTs, is there a baseline captured on similar information? Without a baseline, how could the comparison be done to say whether such a method of information would improve the participation in CT?

Lines 46-47, page 1 – the Discussion/Conclusion section of the abstract that “The initial impression of CTs was quite poor in this socioeconomically disadvantaged sample, but improved after the brief information note on CTs was provided”. Where is the corresponding data for this? Any baseline assessment percentages and comparison with the post test?

Lines 95-98, page 4 – How did the participant selection happen? Was there any particular method of sampling used to select the participants?

Lines 312-314, page 12 – “In our study, providing individuals with brief pertinent information helped to improve their general impression of CTs and, to some extent, their willingness to participate in them” – does it corresponds to the tabular form or textual form?
